# Teeth and Covariates: Association with Risk of Falls

**DOI:** 10.1155/2018/7127209

**Published:** 2018-06-24

**Authors:** Shivani Kohli, Aaron Lam Wui Vun, Christopher Daryl Philip, Cassamally Muhammad Aadil, Mahenthiran Ramalingam

**Affiliations:** ^1^Department of Prosthodontics, Faculty of Dentistry, MAHSA University, Selangor, Malaysia; ^2^Faculty of Dentistry, MAHSA University, Selangor, Malaysia

## Abstract

**Purpose:**

Falls occur commonly in geriatric populations and undesirably influence their life, morbidity, and mortality. The aim of this study was to analyze the association between the number of teeth present among the elderly population and covariates in relation to the risk of falls.

**Materials and Methods:**

This study was conducted at various old age homes in the Klang Valley region of Malaysia involving the geriatric population aged 60 years and above. A detailed questionnaire consisting of sociodemographic data including sex, age, household income, and dental variables such as the number of teeth and chewing difficulty was obtained. The Tinetti test (TT) was used to evaluate the patients' ability to walk, to maintain postural balance, and to determine their risk of falling. The short version of the Geriatric Depression Scale was used to assess depression among the participants, and the Barthel Scale was used to analyze the subject's ability to perform the activities of daily living (ADL).

**Results:**

Statistically significant association was observed in relation to the number of teeth present and risk of falls (*p* < 0.05). Subjects who had 19 teeth or less in total had moderate to highest risk of falls (*p*=0.001) in comparison with subjects who had 20 teeth or more. Those aged 70 years and above showed the highest risk of falls (*p*=0.001) in comparison with the subjects aged between 60 and 69 years. Subjects with depression (*p*=0.03) and presence of illness related to fall showed statistically significant difference (*p*=0.001) in comparison with those who did not suffer from the same. Compromised ADL (*p*=0.001) (which included ability to perform several tasks like indoor mobility, climbing stairs, toilet use, and feeding) and low monthly income (*p*=0.03) was also observed among subjects who had higher risk of falls.

**Conclusion:**

According to the results achieved, there was a high statistically significant association observed between the number of teeth present, age, depression, ADL, and presence of illness in relation to the risk of falling among the geriatric population. Henceforth, oral rehabilitation of elderly patients with less number of teeth may reduce their risk of falls.

## 1. Introduction

According to the World Health Organization (WHO), falls are defined as “inadvertently coming to rest on the ground, floor or other lower level, excluding intentional change in position to rest in furniture, wall or other objects” [1]. Falls have been reported to be a common problem among older people and can be the cause of morbidity and mortality among the later [2]. Incidence of falls of older people is increasing with increasing age and with increasing frailty and dependency. Elderly residents staying in the old age home are approximately three times more prone to falling in comparison with those staying in the community; such falls might even result in bone fracture or hospital admission [3].

Fall incidents may result in hip fracture, joint dislocations, brain injury, facial fracture (head injuries), lower extremity fracture, forearm/wrist fracture, humeral fracture, rib/scapular fracture, severe lacerations, and other soft tissue injuries [4, 5]. Subsequently, falls may restrict daily activities such as bathing, feeding, or dressing and increase the risk of admission to a nursing home [6]. Falls also increase the use of long-term medical services and account for significant care costs [4].

Risk factors for fall injuries have been identified by a number of studies which include older age, white race, arthritis, cerebrovascular disease depression, history of falls, and mainly impairment of muscle strength and balance [7–9]. In view of these risk factors, fall prevention programs have been carried out in the past; however, as they were ineffective, additional risk factors must be investigated [10].

The number of elderly people within a population has been reported to be rising in developing countries including Malaysia [11]. Demographers have estimated that, by the year 2020, almost 10% of Malaysian population will be 60 years and above [12] and aging may also have a detrimental effect on the oral tissues and functions [13]. The elderly are at higher risk of chronic diseases such as dental infections, benign mucosal lesions, xerostomia, and oral candidiasis wherein tooth loss due to caries or periodontal disease has been generally reported as the most common oral condition [14].

Gangloff has investigated the relationship of dental occlusion on gaze and posture stabilization in order to maintain balance and proprioception [15]. Proprioception of the mandible includes the masticatory muscular system and dentoalveolar ligaments (innervation from the trigeminal nerve) which provide sensory afferent input to the central nervous system via vestibular and visual receptors [16]. Alteration in chewing due to lesions in the masticatory muscles or dentoalveolar ligaments could also result in postural imbalances [15]. The effect of different mandibular positions (altered by occlusal collapse) on body equilibrium has also been investigated in the past [17]. Urbanowicz stated that the changes in vertical dimension of occlusion could cause change in head and neck posture [18]. Okuyama et al. associated the loss of dental occlusion with balance function and decline in lower extremities dynamic strength which was the prerequisite for neuromuscular capacity to prevent fall [19]. Several studies have reported the influence of occlusal condition on motor performances and muscle strength of the extremities [20]. However, whether or not the loss of teeth actually predicts the risk of falls is largely unknown. Therefore, the aim of this study was to investigate the influence of the number of teeth present and its covariates in relation to the risk of falls among the elderly aged 60 years and above within Klang Valley region, Malaysia.

## 2. Materials and Methods

This cross-sectional study was conducted over a period of 6 months involving the elderly aged 60 years and above residing in the various cities and towns of the Klang Valley region which was densely populated with a population of approximately 6 million people. The Klang Valley region consists of Wilayah Persekutuan Kuala Lumpur, Wilayah Putrajaya, and subdistricts of Selangor state (Gombak, Hulu Langat, Sepang, Petaling, and Klang).

The study was conducted through one-on-one interview technique, and different tests were performed by the participants which were assessed by the evaluator. Informed consent forms were provided to the participants (either verbal or written) prior to interviews. Subjects aged 60 years and older, both genders, and from any racial background in the Klang Valley region were included. Subjects suffering from physical or cognitive disabilities and those who could not verbally communicate or refused to participate were excluded from the study. The time taken for the interview was based on how fast the participants could respond to the specific question and their capability to perform the physical test; it took approximately 30 minutes per participant to conduct the study. Ethical clearance was obtained from the MAHSA Research Review Committee prior to the commencement of this research.

### 2.1. Study Sample and Recruitment

The sample size was calculated using sample size formula for finite population. The present geriatric population aged 60 and above within the Klang Valley was estimated to be 395276 people according to the latest reports of the Department of Statistics, Malaysia Official Website updated in 2011. The prevalence of home injuries among the elderly in Malaysia was reported as *p*=5.8%  and  95% confidence intervals (CI) in accordance with the study conducted among the elderly people in Malaysia by Lim et al. in 2013 [21].

Normal deviation corresponding to 95% CI [*t*] was set as 1.96, and the absolute error [*d*] was set to 5%. *q* is [1 − *p*]. The total geriatric sample size was estimated to be 77, which was reconfirmed with RAOSOFT calculator, an online calculator used to calculate sample size estimation:

(1) $$\begin{array}{c} \frac{Nt^{2}pq}{d^{2}\left[N-1\right]+t^{2}pq}. \end{array}$$

Convenience sampling technique was used for data collection. Old age homes in the Klang Valley regions were identified; potential participants were initially screened according to eligibility criteria. The interview was conducted using the English, Malay, or Chinese version of the study information based on the participant's language preference.

### 2.2. Study Variables and Questionnaire

A detailed questionnaire consisting of sociodemographic data including sex, age, household income, and dental variables such as the number of teeth present were obtained from the participants by the interviewer. The number of teeth present were observed by the interviewer and categorized into 4 groups: subjects having 20 natural teeth or more, 19 natural teeth or fewer with dentures, 19 natural teeth or fewer without dentures, and absence of teeth as missing.

The participants' ability to masticate was ascertained by asking questions such as “Can eat everything?”, “Can eat most food?”, “Cannot eat most food?”, or “Cannot eat at all?” Their responses were received and evaluated accordingly.

There are studies in the past showing association of falls with sex [7, 21], age [4, 7], activities of daily living [1, 21, 22], depression [4], personal health [21], and movement and socioeconomic factors [23]. Hence, all these parameters were also analyzed in this study. Self-reported current medical conditions such as stroke, osteoporosis, joint disease, neuralgia, and fracture were also recorded as conditions responsible for presence of illness related to fall.

The Tinetti test (TT) was used to evaluate patients' ability to walk, maintain postural balance, and determine their risk of fall [24]. Firstly, the participants were asked to perform the gait component of the test wherein the evaluator walked closely behind the subject to evaluate any abnormality in the gait (steppage) and drift, following which the balance component was executed wherein they performed standing, sitting, and other different actions. Scores of both the components were totaled to determine the level of dependence and risk of falls. The subjects with the highest risk of falls obtained the lowest scores (≤18); moderate risk consisted of people with scores of 19–23 points, which reflected moderate dependence and falling risk; and the group with minimal risk had scores of ≥24 points. To assess depression among the participants, the short version of the Geriatric Depression Scale with fifteen questions constructed for simple answering by means of a straightforward yes/no format was used. It was classified into three groups: 0–4 (no depression), 5–9 (mild depression), and 10–15 (moderate to severe depression). The Barthel Scale was used to analyze subjects' ability to perform the activities of daily living (ADL) which included several tasks like indoor mobility, climbing stairs, toilet use, and feeding [24]. Accordingly, the subjects were categorized as “dependent” or “independent” based on their performances.

### 2.3. Statistical Analysis

Data obtained were tabulated and analyzed using Statistical Package of Social Science version 22, with significance level of 0.05 and 95% confidence intervals. To test the association between the variables and risk of falls, Pearson's chi-square test and Fisher's exact test were employed. Additionally, all covariates were analyzed individually with the risk of falls. Univariate analysis was done to study the relationship between all the variables and risk of fall.

## 3. Results

Out of the 154 participants encountered, 110 were eligible to participate in the study, while others were disqualified according to the exclusion criteria. In order to calculate the *p* value together with the odds ratio and 95% confidence interval for univariate analysis, the total number of teeth present were grouped into subjects having 20 natural teeth or more with or without dentures and 19 natural teeth or less with or without dentures, while the age was grouped into 70 years and above and 60 to 69 years.

Table 1 shows the master chart of the number of teeth present and covariates in relation to the risk of fall wherein 36 (32.73%) out of 110 respondents reported having high risk of fall. It also showed that subjects with less number of teeth, chewing difficulty, older age, females, presence of illness, compromised daily activity, and low monthly income were associated with the risk of falling.

Table 2 shows univariate associations of the number of teeth present and covariates with risk of falls. Subjects having 19 natural teeth or less with or without using dentures have moderate to highest risk of falls (OR 0.27, 95% CI 0.12 to 0.58, *p*=0.001) compared with those having 20 natural teeth or more with or without dentures. Those aged 70 years and above showed a significant result (OR 5.92, 95% CI 2.59 to 13.52, *p*=0.001). Participants with depression showed positive association with risk of falls compared to those without depression (OR 0.25, 95% CI 0.06 to 0.98, *p*=0.03). Subjects with presence of illness related to fall also showed statistically notable relationship (OR 6.87, 95% CI 2.13 to 22.15, *p*=0.001). Activity of daily living also had a significant relation to risk of fall (OR 7.25, 95% CI 2.64 to 19.89, *p* < 0.001). The monthly household income was also recorded to be a crucial factor in relation to the risk of fall (OR 3.32, 95% CI 1.00 to 10.93, *p*=0.03). There is no significant association observed between risk of fall and chewing difficulty, sex, use of drugs, and self-evaluated health.

## 4. Discussion

This was a pilot study to evaluate relationship between remaining teeth and risk of falling among Malaysian elderly population. The present study showed that there was an association between the total number of teeth present and risk of falls. Subjects with 19 or fewer teeth (teeth include both natural and artificial) had a considerably high risk of falls compared to those with more than 20 teeth along with other various extraneous variables such as age, physical health, and household income.

These findings were compatible with the longitudinal cohort study conducted by Yamamoto et al. among Japanese population aged 65 years and above which showed that participants with 19 or lesser number of teeth who were not using partial or complete dental prosthesis (dentures) had more chances of falling compared to people having 20 or more number of teeth [25]. Another longitudinal study by Yoshida et al. [26] conducted among 146 elderly with dementia showed that subjects with functionally inadequate dental status had higher significance of frequent falls compared to those with functionally adequate occlusion composed of natural teeth, denture, or both. They also discovered that falling risk among the geriatric population having 19 remaining teeth or less in total will not increase even if they wear dentures. Such outcomes propose that poor occlusion due to the loss of teeth and without replacing will increase the risk of falling among the elderly subjects with 19 or lesser number of teeth.

There are several possible pathways that relate dentition and incidence of falls, as it is known that falls occur due to loss of balance [22, 27, 28]. Balance is gained through various types of sensory information emanating from proprioceptive systems. The stomatognathic system (maxilla and mandible, dental arches, salivary glands, nervous and vascular supplies, temporomandibular joint, and masticatory muscles) may affect muscular function in other parts of the body, range of movement, and balance control [15, 23, 29–31]. Dental occlusion does influence postural stabilization through sensory afferent input from the ligaments around the teeth and the masticatory system. Consequently, improper occlusion may reduce the proprioception and interfere with the head stabilization [19]. Miyaura et al. in 2000 also showed that denture use improves postural balance [32].

In the present study, self-reported chewing difficulty was not associated with the risk of falls. This result was similar to the study conducted by Yamamoto et al. in 2012 [25] but was in contrast with another study conducted by Takata et al. [31] in 2004 which showed significant association between chewing ability and balance, as self-reported chewing ability can be very subjectively answered based on the type of food intake (soft food or solid food).

Brito et al. in 2014 reported the occurrence of falls was accompanied with depressive symptoms and disturbances in balance as recorded in the present study [33]. Fall happens more often to people with lower functional status which was similar to the results obtained in this study [34]. For the other covariates discussed in this present study, the gender was not the risk factors for fall which is in accordance with existing scientific guidelines for prevention of falls [22, 35]. Self-reported finding such as general health was the limitation of the present study. Future study with a larger sample size would be more helpful to confirm the impact of different variables in relation to the risk of fall among the elderly.

## 5. Conclusion

The purpose of this study was to evaluate the importance of the presence of teeth among the geriatric population, and care should be taken to preserve them in order to reduce their risk of falls. Highly statistically significant association was observed between the number of teeth present and the risk of fall; hence, dental health education including taking care of own teeth, yearly dental checkup, and proper use of denture may help in preventing falls. Among the covariates, statistically significant association was seen between age, depression, presence of illness related to fall, ADL, and household income in relation to the risk of fall. Retrospectively, people with high risk of falling may be identified through dental checkup, and oral rehabilitation can be done to reduce their risk of falls. Further studies testing the influence of maintaining oral health and denture usage on prevention of falls should be carried out.

## Figures and Tables

**Table 1:** Master chart of number of teeth present and covariates in relation to the risk of fall.

Master chart	Total	Tinetti test (TT)		
		Lowest risk of falls	Moderate risk of falls	Highest risk of falls
*Total number of teeth present*				
≥20 natural teeth	28	17 (60.7)	5 (17.9)	6 (21.4)
≤19 natural teeth with dentures	34	25 (73.5)	3 (8.8)	6 (17.6)
≤19 natural teeth without dentures	42	13 (31.0)	7 (16.7)	22 (52.4)
Missing	6	4 (66.7)	0	2 (33.3)

*Chewing difficulty*				
Can eat everything	41	25 (61.0)	10 (24.4)	6 (14.6)
Can eat most foods	39	21 (53.8)	0	18 (46.2)
Cannot eat most foods	28	12 (42.9)	4 (14.3)	12 (42.9)
Cannot eat at all	2	1 (50)	0	1 (50)

*Sex*				
Male	48	26 (54.2)	6 (12.5)	16 (33.3)
Female	62	33 (53.2)	8 (12.9)	21 (33.9)

*Presence of illness related to fall*				
Yes	21	4 (19.1)	4 (19.1)	13 (61.9)
No	89	55 (61.8)	10 (11.2)	24 (27.0)

*Activity of daily living*				
Dependent	29	6 (20.7)	5 (17.2)	18 (62.1)
Independent	81	53 (65.4)	9 (11.1)	19 (23.5)

*Use of drugs*				
Yes	73	35 (47.9)	11 (15.1)	27 (37.0)
No	37	24 (64.9)	3 (8.1)	10 (27.0)

*Self-evaluated health*				
Excellent	8	2 (25)	2 (25)	4 (50)
Good	62	39 (62.9)	9 (14.5)	14 (22.6)
Fair	39	17 (43.6)	3 (7.7)	19 (48.7)
Poor	1	1 (100)	0	0

*Monthly household income (Malaysian ringgit)*				
<500	93	46 (49.5)	13 (14)	34 (36.6)
500–999	4	4 (100)	0	0
1000–1999	5	3 (60)	1 (20)	1 (20)
>2000	8	6 (75)	0	2 (25)

**Table 2:** Univariate associations of the number of teeth present and covariates with risk of falls.

Covariates	Total	Risk of falls (TT)		*p* value	OR	95% CI
		Lowest risk of falls	Moderate to highest risk of falls			
*Total number of teeth present*						
20 teeth or more with/without denture	62	42	20	0.001	0.26	0.12–0.58
19 teeth or less with/without denture	48	17	31			

*Age*						
70 years old and above	53	17	36	<0.001	5.92	2.59–13.52
60 to 69 years old	57	42	15			

*Sex*						
Male	48	26	22	0.92	0.9629	0.45–2.05
Female	62	33	29			

*Depression*						
No	98	56	42	0.03^*∗*^	0.25	0.06–0.98
Mild to severe	12	3	9			

*Presence of illness related to fall*						
Yes	21	4	17	0.001	6.87	2.13–22.15
No	89	55	34			

*Activity of daily living (ADL)*						
Dependent	29	6	23	<0.001	7.25	2.64–19.89
Independent	81	53	28			

*Use of drugs*						
Yes	73	35	38	0.09	2.00	0.88–4.53
No	37	24	13			

*Self-evaluated health*						
Healthy	70	41	29	0.16	0.57	0.26–1.26
Not very healthy	40	18	22			

*Monthly household income (Malaysian ringgit)*						
500 or less	93	46	47	0.03	3.32	1.00–10.93
501 or more	17	13	4			

^*∗*^Calculated using Fisher's exact test.
